# Prevalence of hospital-acquired infections in the university medical center of Rabat, Morocco

**DOI:** 10.1186/1755-7682-5-26

**Published:** 2012-10-02

**Authors:** Rachid Razine, Abderrahim Azzouzi, Amina Barkat, Ibtissam Khoudri, Fadil Hassouni, Almontacer Charif Chefchaouni, Redouane Abouqal

**Affiliations:** 1 Department of Public Health, Faculty of Medicine and Pharmacy of Rabat, Rabat, Morocco; 2 Laboratory of Biostatistics, Clinical Research and Epidemiology, Faculty of Medicine and Pharmacy of Rabat, Rabat, Morocco; 3 Surgical Intensive Care Unit, University Medical Center of Rabat, Rabat, Morocco; 4 National Center of Neonatology and Nutrition, Children’s Hospital, University Medical Center of Rabat, Rabat, Morocco; 5 University Medical Center of Rabat, Rabat, Morocco; 6 Medical Emergency Department, University Medical Center of Rabat, Rabat, Morocco

**Keywords:** Hospital-acquired infections, Prevalence survey, Morocco

## Abstract

**Background:**

The aims of this study were to determine the hospital-acquired infections (HAI) prevalence in all institutions of Rabat University Medical Center, to ascertain risk factors, to describe the pathogens associated with HAI and their susceptibility profile to antibiotics.

**Materials and methods:**

Point-prevalence survey in January 2010 concerning all patients who had been in the hospital for at least 48 hours. At bedside, 27 investigators filled a standardized questionnaire from medical records, temperature charts, radiographs, laboratory reports and by consultation with the ward’s collaborating health professionals. Risk factors were determined using logistic regression.

**Results:**

1195 patients involved, occupancy rate was 51%. The prevalence of HAI was 10.3%. Intensive care units were the most affected wards (34.5%). Urinary tract infection was the most common infected site (35%). Microbiological documentation was available in 61% of HAI. *Staphylococcus* was the organism most commonly isolated (18.7%) and was methicillin-resistant in 50% of cases. In multivariate analysis, risk factors associated with HAI were advanced age, longer length of hospital stay, presence of comorbidity, invasive devices and use of antibiotic use.

**Conclusion:**

HAI prevalence was high in this study. Future prevention program should focus on patients with longer length of stay, invasive devices, and overprescribing antibiotics.

## Background

Hospital-acquired infections (HAI) are a major public health problem all over the world, but particularly in developing nations [1]. The surveillance of HAI is regarded as an essential part of infection control and prevention. Despite the fact that prevalence studies have well known disadvantages, they can be a useful part of an effective surveillance system and help to identify areas for further investigations [2,3]. Resources spent on health care in order to control HAI are variable following the countries concerned; some nations have limited resources, while others may have largest ones. Furthermore, repeated comparable prevalence surveys can provide information regarding the evolution of HAI trends [4,5]. Prevalence studies can be particularly useful where financial resources and qualified personnel are in short supply, because prevalence surveys can be conducted quickly and without sophisticated techniques. Meager and often inconsistent survey data on HAI are common in developing countries [6].

In Morocco, the prevention of HAI has begun to arouse interest in recent years and some hospitals have developed their own prevention programs, although there is still no national legislation requiring the reporting of all HAI cases. The first Moroccan national survey of HAI was conducted in 1994 and found a prevalence rate of 9%, but no data are published about this study. Then, few single center studies have been published in our context [7,8]. In Rabat, an earlier study has been published in 2007. This study reported an HAI prevalence rate of 17.8%, but was conducted only in a single hospital of the whole Rabat University Medical Center [8].

This paper presents data obtained about HAI from a prevalence study conducted in all hospitals of the Rabat University Medical Center. The aims of this study were: a) to determine the HAI prevalence and ascertain the associated risk factors b) to describe the pathogens associated with HAI and their susceptibility profile to antibiotics.

## Methods

### Setting

This study was designed as a point-prevalence survey performed between January 13 and 15 2010. It was conducted in all wards of eight hospitals of Rabat University Medical Center (Table 1). Rabat is the political and administrative capital of Morocco and the second largest city in the country. Rabat University Medical Center is the largest hospital complex in Morocco and in North-Africa. This teaching medical center is a 2 535-bed tertiary-stage hospital. It comprises 10 hospitals and admitted patients from all specialties and all ages from all regions of Morocco. The average occupancy rate is around 73%.

**Table 1:** Institutions of Rabat University Medical Center included in the study

**Institution**	**Specialties**	**Functional bed capacity**
***Ibn Sina Hospital***	All except neurology, Oto-Rhino-Laryngology , ophthalmology, Gynecology and obstetrics, Pediatrics, Oncology and Rheumatology	959
***Hospital of cephalic specialties***	Neurology, Neurosurgery, ophthalmology, Oto-Rhino-Laryngology	321
***National Center for Reproductive Health***	Gynecology and obstetrics	80
***Pediatric hospital***	Pediatrics	407
***National Institute of Oncology***	Oncology	216
***Moulay Youssef Hospital of Phtisiology***	Phtisiology	193
***Souissi maternity***	Gynecology and obstetrics	269
***Eyachi hospital of Rheumatology***	Rheumatology	72

### Study population

All the patients present in the University Medical Center in the 48 hours or greater preceding the survey were investigated. Patients from two institutions of Rabat University Medical Center: the dental treatment clinic and psychiatric hospital were excluded. Patients hospitalized in emergency departments were also excluded. The study was approved by the Rabat University Medical Center Ethics Committee, and informed consent was obtained from all participants.

### Data collection

Twenty seven investigators (physicians and nurses) participated in the study. They all received a training course of half a day before the study and were directed by a principal investigator. They collected data from clinical records, temperature charts, radiographs, laboratory reports and consultation with the ward’s collaborating health professionals. Physical examinations were conducted by the ward’s physician in the presence of the investigator in order to have information about patient’s current status. Furthermore, evaluation notes on surgical wounds were examined from the doctor’s notes. During the visit to hospitalized patients, the investigators (physicians and nurses) were seeking for signs of HAI for each patient in collaboration with a referent hygiene agent which is a nurse. Infections affirmed clinically and / or microbiologically were selected. A follow-up of 48 or 72 hours was sometimes necessary to obtain the results of complementary examinations during the study day and likely to confirm (or disprove) the HAI. The 27 investigators were distributed among the different care units. Some of them have visited multiple care units with the respect of the rule that a care unit should be surveyed in the same day.

Data collected for each patient were: age, gender, admission date, ward type, duration of hospital stay, underlying pathologies including diabetes, and the American Society of Anesthesiologists (ASA) score [9]. Patients were classified into three categories according to the severity of illness as proposed by McCabe and Jackson [10]. Conditions of immune deficiency as defined by Knaus et al. [11] were noted. Surgical operations within the past 30 days and their classification following Altemeier [12] were also recorded. Exposures to invasive devices (urinary catheter, central intravascular catheter, peripheral intravascular catheter, and mechanical ventilation) on the day of, or during the 7 days before the survey were noted. The HAI occurrence, HAI site, micro-organisms responsible for HAI, antimicrobial susceptibility patterns when available, type and reasons for antibiotic treatment were collected.

### Definitions of hospital-acquired infection

The criteria of the Centers for Disease Control and Prevention (CDC) Atlanta, USA, were used to define HAI [13,14]. An infection was defined as a nosocomial infection when it originated in the hospital environment, was neither present nor incubating at the time of admission to the hospital, and appeared 48 hours or more after admission. The HAI was classified as urinary tract infection, surgical wound, lower respiratory tract infection, bloodstream, skin and soft-tissue infections, catheter related infection and others.

An active HAI was defined when antimicrobial treatment was still being given on the day of the survey.

### Statistical analysis

Categorical variables were expressed as percentages, and continuous variables were expressed as means ± SD or median (interquartile range). Prevalence of HAI and the prevalence of infected patients were calculated. The 95% confidence intervals (CIs) were estimated. To study risk factors, univariate analyses were first performed using simple logistic regression. Variables with P values < 0.20 in the univariate analysis were tested in the multivariate analysis. Multivariable logistic regression model with forward stepwise variable inclusion was used. Adjusted odds ratios (OR) and their 95% CIs were derived. A *p* value of 0.05 or less was considered to be statistically significant. Data were analyzed using the statistical software SPSS version 13.0 (SPSS; Chicago, IL, USA).

## Results

### Patient and hospital characteristics

**Figure 1:**
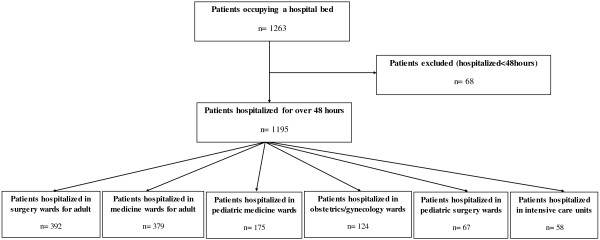
Flow-chart of the patients included in the study and hospitalization wards at the day of survey.

A total of 1263 patients occupied a bed at the day of survey. The bed occupancy rate was 51%. From total patients, 1195 had been hospitalized for over 48 hours. We excluded 68 patients because they were admitted on the day of the study. Sixty three wards were visited in the eight institutions of the University Medical Center included in the survey. Of the screened patients, 392 patient (32.8) were hospitalized in surgery wards for adult, 379 (31.7) were hospitalized in medicine wards for adult, 175 (14.6) in pediatric medicine wards, 124 (10.4) in obstetrics and gynecology wards, 67 (5.6) in pediatric surgery wards, and 58 patients (4.9) were hospitalized in intensive care units (ICUs) (Figure 1).

**Figure 2:**
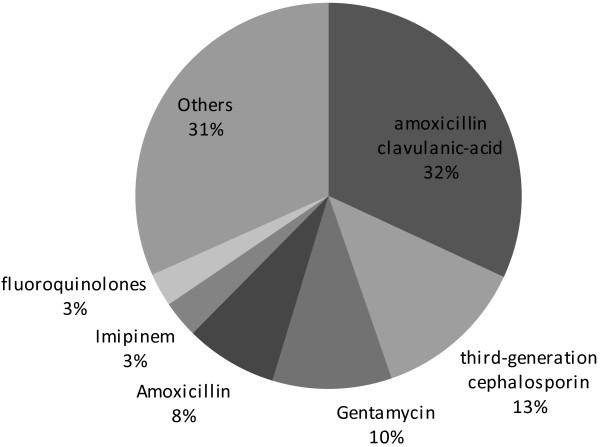
Antibiotics most commonly used.

The characteristics of the study population and extrinsic exposures are noted in Table 2. Of the 1195 patients included in the survey, 600 (50.2%) were women and 595 (49.8%) were men. Mean age was 35.8 ± 23.2 years. Median hospital length of stay from admission to the study day was 10 (5–20) days. Immune deficiency was reported in 16.3% of patients. A total of 240 patients (20.1%) underwent surgical intervention during the month before the survey. Surgical wounds were clean in 58.6%, clean-contamined in 30.5%, contamined in 8.6%, and dirty in 2.4%. A total of 273 patients (23.1%) had a vascular catheter and 114 (9.5%) had urinary catheter on the day of, or during the 7 days before the survey. At the time of the study, 392 (32.8%) patients were receiving antimicrobial drugs. They were curative in 76% and prophylactic in 24% of cases. Figure 2 illustrates the categories of antibiotics administered.

**Table 2:** Patient characteristics and extrinsic exposures

	**Number of patients**	**Percentage**
**Age** (years)		
≤ 15 ans	283	23.7
16 - 40	387	32.4
41 - 60	339	28.4
> 60	186	15.6
**Gender**		
Female	600	50.2
Male	595	49.8
**Provenance**		
home	932	78.0
Hospital	263	22.0
**MacCabe index**		
Non fatal disease	742	62.1
Ultimately fatal disease	394	33.0
Rapidly fatal disease	59	4.9
ASA* grade		
0	4	0.3
1	533	44.6
2	183	15.3
3	90	7.5
4	10	0.8
5	0	0
unspecified	375	31.4
**Immune deficiency**		
Yes	195	16.3
No	1000	83.7
**Diabetes**		
Yes	101	8.5
No	1094	91.5
**Surgery**		
Yes	240	20.1
No	955	79.9
**Intravascular catheter**		
Yes	276	23.1
No	919	76.9
**Urinary catheter**		
Yes	114	9.5
No	1081	90.5
**mechanical ventilation**		
Yes	14	1.2
No	1181	98.8
**Antimicrobials**		
Yes	392	32.8
No	803	67.2
**Length of hospital sty** (days)		
≤ 5	341	28.5
6 - 10	317	26.5
11 - 20	245	20.5
> 20	292	24.4

*American Society of Anesthesiologists.

### Prevalence, sites and pathogens of infections

There was a total of 123 HAI in 116 patients. Thus, the overall prevalence of infections which is the mean prevalence of all hospitals of Rabat University Medical Center was 10.3% (95% CI, 8.6%–12%). The prevalence of infected patients was 9.7% (95% CI, 8% - 11.4%). Among 116 patients with HAI, 109 (94%) had a single infection and 7 (6%) had two infections. The prevalence of HAI was highest in ICUs (34.5%) and lowest in pediatric surgery (1.5%) (Table 3). The frequency of urinary tract infections was the highest (35%) followed by surgical wound infection (29.3%), lower respiratory tract infection (10.6%), bloodstream infection (8.1%), skin and soft tissue infection (5.7%), catheter related infection (4.9) and others (6.4). The prevalence of HAI at Ibn Sina hospital, the largest institution in the University Medical Center, was 10.1%.

**Table 3:** Prevalence of Hospital Acquired Infection (HAI) in different wards

**Wards**	**Prevalence of HAI**		**95% CI**
	**Number of patients**	**Percentage**	
Intensive care units	20	34.5	31.8 – 37.2
Obstetrics and Gynecology	15	12.1	10.3 – 13.9
Surgery for adults	53	13.5	11.6 – 15.4
Pediatric medicine	17	9.7	8.0 – 11.4
Medicine for adults	17	4.5	3.3 – 5.7
Pediatric Surgery	1	1.5	0.8 – 2.2

**Figure 3:**
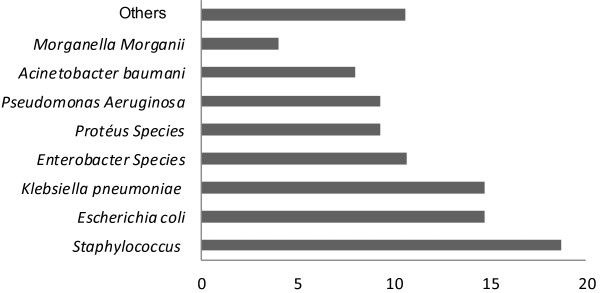
Frequency of organisms isolated.

Among the 123 episodes of HAI, 75 micro-organisms were isolated (61%). The predominant micro-organisms were *staphylococcus* (18.7%) followed by *Escherichia coli* (14.7%) and *Klebsiella pneumoiae* (14.7%) (Figure 3). The site of infection most frequently affected by *Staphylococcus* was urinary tract (42.9%). Methicillin-resistant strains accounted for 50% of isolated *Staphylococcus. Escherichia coli* was resistant to fluoroquinolones in 27% of cases and to amoxicillin-clavulanic acid in 36% of cases.

### Risk factors

In univariate analysis, patient characteristics and exposure to invasive devices increasing the risk of HAI were: McCabe index, undergoing surgery, longer duration of hospital stay, exposure to intravascular or urinary catheter, mechanical ventilation and use of antimicrobials (Table 4).

**Table 4:** Intrinsic and extrinsic risk factors for Hospital Acquired Infection: univariate and multi-variate analysis (logistic regression)

	**Infected patients (%)**	***Univariate analysis***			***Multivariate analysis***		
		OR*	**95% CI**	***p*value**	OR*	**95% CI**	***p*value**
**Age** (years)							
≤ 15	8.5	1.07	0.6-1.9	0.815	1.03	0.47-2.28	0.939
16 - 40	9.6	1.22	0.7-2.1	0.450	1.24	0.67-2.57	0.65
41 - 60	8.0	1			1		
> 60	15.1	2.05	1.2-3.6	**0.012**	2.71	1.21-4.95	**0.006**
**Gender**							
Female	9.3	1					
Male	10.1	1.09	0.7-1.6	0.66			
**Length of hospital stay** (days)							
≤ 5	3.5	1			1		
6 - 10	9.5	2.87	1.4-5.7	**0.003**	3.79	1.68- 8.93	**0.001**
11 - 20	11	3.40	1.7-6.8	**0.01**	4.40	1.83-10.61	**0.001**
> 20	16.1	5.26	2.7-10.1	**<0.001**	9.6	3.63-18.51	**<0.001**
**Provenance**							
Home	8.5	1			1		
Hospital	1.1	1.77	1.2-2.7	0.07	1.45	0.84-2.50	0.18
**MacCabe index**							
Non fatal disease	6 .3	1			1		
Ultimately fatal disease	14.5	2.5	1.7-3.8	**<0.001**	2.01	1.21-3.36	**0.007**
Rapidly fatal disease	20.3	3.8	1.9-7.6	**<0.001**	2.50	0.99-6.27	0.051
**Immune deficiency**							
No	10	1					
Yes	8.2	0.80	0.46-1.40	0.44			
**Diabetes**							
No	6.7	1			1		
Yes	17.4	1.57	0.86-2.85	0.14	0.98	0.44-2.14	0.95
**Surgery**							
No	6.9	1			1		
Yes	20.8	3.55	2.37-5.29	**<0.001**	1.06	0.61-1.85	0.83
**Intravascular catheter**							
No	6.3	1			1		
Yes	21.0	3.95	2.67-5.86	**<0.001**	1.75	1.0-3.1	**0.048**
**Urinary catheter**							
No	5.5	1			1		
Yes	50	17.32	11.03-27.2	**<0.001**	9.84	5.16-17.55	**<0.001**
**Mechanical ventilation**							
No	9	1			1		
Yes	71.4	25.35	7.82-82.2	**<0.001**	3.43	0.70-17.00	0.131
**Antimicrobials**							
No	4.4				1		
Yes	20.7	5.72	3.76-8.78	**<0.001**	5.69	3.37-9.61	**<0.001**

* Odds Ratio.

In the stepwise forward logistic regression, the variables found to be significantly associated with HAI were: Older age > 60 years (OR = 2.71; 95% CI = 1.21-4.95), longer duration of hospital stay (from 6 to10 days: OR = 3.79; 95% CI = 1.68- 8.93, from11to 20 days: OR = 4.40; 95% CI = 1.83-10.61, and >20 days: OR = 9.6; 95% CI = 3.63-18.51), McCabe index (ultimately fatal disease OR = 2.01; 95% CI = 1.21-3.36), intravascular catheter (OR = 1.75; 95% CI = 1.0-3.1), urinary catheter (OR = 9.84; 95% CI = 5.16-17.55) and antimicrobial use (OR = 5.69; 95% CI = 3.37-9.61).

## Discussion

In this study, we found an HAI prevalence of 10.3% which is higher than what was found in several countries since 2000: 4.9% in Italy [15], 7.2% in Switzerland [16], 5.4% in Norway [17], 7,2% in Netherland [18] and 5.4% in French [19]. However, this rate was comparable to that reported in some developing countries such as Senegal (10.9%) [20] and Tunisia (17.9%) [21]. However, these comparisons are purely illustrative because the methods used (definitions and types of HAI identified, methods of case finding, exclusion of imported HAI or not) and hospitals, or patients included are different following the surveys.

We believe that the high prevalence rate found in our study can be explained firstly by the absence of a national strategy to prevent HAI in Moroccan hospitals; and secondly by the highly developed healthcare systems that university hospitals offer including invasive medical and surgical procedures. It should also be noted that prevalence studies are certainly less expensive faster and easier to achieve, but the results are subject to seasonal fluctuations and can sometimes coincide with epidemic peaks. Moreover, another limitation of prevalence studies is that the temporal sequence between exposure and health event cannot be confirmed with certainty. Therefore, the association between risk factors and HAIs should be interpreted with caution. Incidence studies may then be better, they are better suited to detect risk factors, and can be privileged when possible [22-24].

The prevalence rate found in Ibn Sina hospital (10.1%) is also high but less important to that reported in the same hospital in 2005 (17.8%) [6]. This decrease is may be due to the efforts of the local HAI control committee instituted in 2005 after the survey. These efforts have focused primarily on educating health professionals and administrators to improve control methods and prevention of HAI especially the methods of washing hands.

The most affected wards in our study were ICUs. This finding is consistent with the literature data [15,25-28] and can be explained by the frequency of severe disease laying in ICUs, the prescription of broad spectrum antibiotics and also use of devices and invasive procedures.

Our study identified three main sites of HAI: urinary tract infection, pneumonia and surgical wound infection. These sites are the most frequently reported in prevalence surveys [8,15,19,28].

The strong association between HAI and urinary catheter should lead to adopt some preventive measures in order to limit the use of urinary catheter for the only absolute necessity.

Surgical site infections appear higher compared with what is reported in several regions of the world [15,17,20,21,29,30]. This fact can be explained on the one hand by the lack of program and institutional procedures for the prevention of HAI in this context and on the other hand, by the fact that our university center includes referral hospitals that receive complex surgical cases from all over Morocco.

This study gave us also the opportunity to describe the bacterial ecology associated with HAI in Rabat University Medical Center. The organisms most frequently isolated were *Staphylococcus*, *Escherichia coli* and *Kliebsiella. Staphylococcus* remains the frequent germ found in most studies [8,15,16]. However, the high percentage of *Escherichia coli* and *Kliebsiella* can be explained by the high frequency of urinary tract infections.

*Staphylococcus* was methicillin-resistant in 50% of cases, while *Escherichia coli* was fluoroquinolone resistant in about 27% of cases and amoxicillin-clavulanic acid resistant in 36% of cases. Because these represent the antibiotics most frequently used in practice, serious problems can be encountered while prescribing those antibiotics. Establishing guidelines for prescribing antibiotics become then a necessity.

In our study, risk factors associated with HAI were: older age, longer hospital length of stay, comorbidity, invasive devices and overprescribing antibiotics. Other studies reported similar results to ours [7,31-33]. Control strategies of HAI should then primarily target these factors, for example: reducing the length of stay, limiting the indications and duration of invasive devices, limiting antibiotic prescriptions especially broad-spectrum ones. Using guidelines of good practice for prescribing antibiotics may also reduce the risk of HAI.

The major limitation of the study is the bed occupancy rate. The bed occupancy rate in our study (51%) was lower than the usual one (around 70%). This fact is may be due to the most important religious celebration of the year (celebration of Aladha) coincided with the study. However, even though the bed occupancy rate may seem week, the distribution of patients following hospitals and wards is similar to what is found in periods of normal rate occupancy. Another limitation of the study is that data were collected by 27 investigators. This may introduce bias in the study. However, to overcome this bias, a training course of half a day was organized before the study in order to standardize the procedures for data collection and reduce as possible the effect of the bias.

## Conclusion

Ultimately, the prevalence of HAI was high in the Rabat University Medical Center. This study represents basic information for future monitoring of HAI and should be repeated periodically. It allowed us to describe the profile of patients at high risk of developing HAI. Thus we believe that the future prevention program should focus on patients with longer length of stay and those with invasive devices. At the institutional level, it is urgent to establish HAI prevention programs and maybe also a national strategy in this way.

Elsewhere, prospective studies are desirable in order to describe more accurately HAI incidence as well as risk factors in each context.

### Key messages

Hospital-acquired infections in Rabat

## Abbreviations

ASA: American Society of Anesthesiologists; CDC: Centers for Disease Control and Prevention; CI: Confidence Interval; HAI: Hospital-acquired infection; ICU: Intensive Care Unit; OR: Odds Ratio.

## Competing interests

The authors declare that they have no competing interests.

## Authors’ contributions

**RR** drafted the manuscript, participated in the acquisition of data, and performed the statistical analysis; **AA** participated in the coordination of the study; **AB** participated in the acquisition of data and participated in the coordination of the study; **IK** helped to draft the manuscript; **FH** participated in the coordination of the study; **ACC** participated in the coordination of the study; **RA** conceived of the study, participated in the design of the study, performed the statistical analysis and interpretation of data, and gave the final approval of the manuscript. All authors read and approved the final manuscript.
